# A Common *OXTR* Risk Variant Alters Regulation of Gene Expression by DNA Hydroxymethylation in Pregnant Human Myometrium

**DOI:** 10.1007/s43032-024-01621-9

**Published:** 2024-06-11

**Authors:** Joshua S. Danoff, Travis S. Lillard, Leslie Myatt, Jessica J. Connelly, Elise N. Erickson

**Affiliations:** 1 Department of Psychology, University of Virginia, Charlottesville, VA USA; 2 Department of Molecular Biology and Biochemistry, Rutgers University, Piscataway, NJ USA; 3 Department of Obstetrics and Gynecology, Oregon Health and Science University, Portland, Oregon USA; 4 College of Nursing, University of Arizona, Tucson, AZ USA

**Keywords:** DNA methylation, DNA hydroxymethylation, Oxytocin receptor, Postpartum hemorrhage, Epigenetic biomarker, Myometrium

## Abstract

Postpartum hemorrhage, or excessive bleeding after birth, is a leading cause of maternal morbidity. A major cause of postpartum hemorrhage is uterine atony, tiring of the uterus which leads to ineffective contractions. Uterine contractions depend on oxytocin signaling in the myometrium, which in turn depends on expression of the oxytocin receptor (OXTR). Both genetic and epigenetic factors related to the oxytocin receptor are associated with risk of postpartum hemorrhage, but a mechanism relating these factors to oxytocin receptor activity in myometrium remains unclear. We report a genetic by epigenetic interaction whereby the relationship between DNA hydroxymethylation and *OXTR* gene expression depends on a common *OXTR* gene variant (rs53576). We also provide evidence that a similar genetic by epigenetic interaction using blood-derived DNA methylation is associated with relevant clinical outcomes: quantity of oxytocin administration and odds for postpartum hemorrhage. These results provide new avenues for predicting how women will respond to pharmacological agents in the prevention and treatment of postpartum hemorrhage.

**Supplementary Information:**

The online version contains supplementary material available at 10.1007/s43032-024-01621-9.

## Introduction

Postpartum hemorrhage, defined as greater than 1000 mL blood loss within 24 h following birth, is among the leading causes of maternal mortality [1, 2]. One major cause of postpartum hemorrhage is uterine atony, which occurs when myometrium does not effectively contract during the third stage of labor [3]. Uterine contractions during the third stage of labor serve to put pressure on the vasculature supplying the myometrium and decidua and stop bleeding. During this stage of labor, uterine contractions are dependent on oxytocin signaling [4]. Oxytocin signaling in the myometrium depends on expression of the oxytocin receptor (OXTR), which is highly upregulated during late pregnancy [5]. We demonstrated that a common genetic variant in the third intron of *OXTR* (rs53576) confers risk for postpartum hemorrhage, particularly in women without other common risk factors [6].

Oxytocin receptor gene expression is regulated by DNA methylation in a region of the gene called MT2 [7, 8]. DNA methylation at this CpG site is associated with reduced gene expression in the brain [8]. Indirect evidence suggests DNA methylation is reduced in myometrium during pregnancy, leading to increased gene expression, though the mechanism is far from clear [7]. We recently provided evidence that *OXTR* DNA methylation in the blood is reflective of DNA methylation in the myometrium [9]. Further, we showed that *OXTR* methylation is associated with higher postpartum blood loss and need for postpartum oxytocin administration, the first line treatment for postpartum hemorrhage [9]. However, how DNA methylation and genetic factors interact in regulating *OXTR* expression or conferring risk for postpartum hemorrhage is not yet established.

Here, using myometrium samples collected from women undergoing unlabored cesarean section, we provide evidence of a genetic by epigenetic interaction regulating *OXTR* expression in pregnant myometrium which may explain differential risk of postpartum hemorrhage associated with SNP rs53576. Further, using data from a sample of participants undergoing vaginal birth, we provide preliminary evidence of such differential risk using blood-derived measurements of DNA methylation.

## Methods

### Matched Myometrium and Blood Tissue Collection (Cohort 1)

Participants and procedures for myometrium collection were previously reported and are summarized below [9]. Participants were available via a pregnancy-related tissue repository managed by Oregon Health and Science University. Power analysis was not used prior to the study to determine sample size. All participants provided written informed consent. Participants ages ranged from 20–40 years (median 34 years) and all were multiparous. Participant BMI ranged from 21.5 to 54.9 (median 29.6). Gestation length at the time of cesarean ranged from 37 weeks to 39.7 weeks (median 39 weeks). 13 participants identified as White and non-Hispanic, 1 participant identified as White and Hispanic, and 1 participant identified as Black and non-Hispanic. Additionally, 1 participant identified as White but did not provide information regarding Hispanic ethnicity, and 1 participant did not self-identify race but did indicate Hispanic ethnicity. Limited clinical information was collected from participants donating myometrial tissue. Cesarean delivery was indicated due to prior cesarean, presence of chronic or gestational hypertension, or choice. Whole blood was collected in EDTA tubes within the hour prior to delivery and was frozen at -80 °C. After birth of the newborn and placenta, a portion of full-thickness myometrium was excised and rinsed in phosphate buffered saline, dissected from serosa and decidua if present, and flash frozen in liquid nitrogen.

### Participants for Oxytocin Administration Outcomes (Cohort 2)

Participants used to compare blood-derived DNA methylation measurements to oxytocin needs during labor were enrolled in a case–control study designed to study how *OXTR* genetic variants are related to postpartum hemorrhage outcomes. Procedures for enrollment in this study and collection of clinical data are reported elsewhere and summarized below [6]. Participants were enrolled from both hospital and community birth settings in the greater Portland, Oregon area. Oxytocin administration was calculated from medical records. Oxytocin administration start and stop times and the timing and duration of titration of oxytocin doses were used to calculate the total number of milliunits (mU) of oxytocin administered during labor. Oxytocin given after birth for the active management of the third stage of labor was also recorded. Between 6–8 weeks after birth, maternal blood samples were obtained for DNA methylation and rs53576 genotype analysis. Demographic information are reported in our previous publication using this sample[9].

### DNA Methylation and rs53576 Genotype Measurements from whole Blood and Myometrium

Procedures for DNA isolation, methylation analysis, and genotyping were previously described and are summarized below [6, 9]. DNA was isolated from whole blood using the QIAamp DNA Mini Kit (Qiagen, Hilden, Germany). DNA was isolated from myometrium in the same manner. DNA was bisulfite converted using MECOV50 kits (Thermo Fisher Scientific, Waltham, USA). Following bisulfite conversion, DNA was amplified using PCR with Pyromark PCR kits (Qiagen) to amplify a region of *OXTR* containing CpG site -934. PCR was completed in triplicate. DNA methylation was measured by pyrosequencing of the PCR product using PyroMark Gold Q24 reagents on a PyroMark Q24 machine (Qiagen). 0% and 100% methylation standards were included as controls.

Genotyping of whole blood samples was completed using PCR with Amplitaq gold 360 PCR reagents on a 9700 GenAmp thermocycler using manufacturer instructions (ThermoFisher Scientific). PCR products were sequenced using a BigDye Terminator v3.1 cycle sequencing kit and the products were analyzed on a 3130XL Genetic analyzer (ThermoFisher Scientific).

Genotyping of myometrium samples was completed by PCR amplifying a region containing the SNP using Pyromark PCR reagents (Qiagen), 0.2 µM of the forward and reverse primers (F: 5’-AAAGGTGTACGGGACATGCC-3’; R: 5-biotin-TTTCCCATCTGTAGAATGAGC-3’), 10 ng genomic DNA as input, and the following cycling conditions: [Step 1: (95 °C/15 min)/1 cycle, Step 2: (94 °C/30 s, 60 °C/30 s, 72 °C/30 s)/45 cycles, Step 3: (72 °C/10 min)/1 cycle, Step 4: 4 °C hold]. PCR product was pyrosequenced on a Q24 machine (Qiagen) with Pyromark Gold Q24 reagents (Qiagen) and sequencing primer 5’-TTCTGTGGGACTGAGG-3’.

### RNA Isolation from Myometrium

Myometrial tissue was crushed using a mortar and pestle over liquid nitrogen and transferred to a glass tissue grinder. 1 mL TRI reagent (Thermo Fisher Scientific) was added and the tissue was homogenized. RNA was isolated according to manufacturer protocol. After phase separation with BCP, the sample was centrifuged at 12,000 g for 15 min at 4 °C. The aqueous phase was transferred to a new tube and RNA was precipitated with isopropanol. After a 10 min room temperature incubation, RNA was pelleted by centrifuging at 12,000 g for 8 min at 4 °C. RNA was washed once with 75% ethanol and centrifuged again to collect the pellet. The ethanol was removed from the pellet and RNA was air-dried for 3 min. The RNA was then reconstituted in water and incubated at 55 °C for 10 min to solubilize. RNA was stored at -80 °C until sequencing.

### RNA Sequencing and Analysis

RNA sequencing was performed by the OHSU Massively Parallel Sequencing Shared Resource. RNA quality was assessed using Agilent Bioanalyzer 2100. RIN scores ranged from 3.2 to 8.5 (median 6.5). 100 ng RNA was used as input for library prep with ribosomal and globin RNA depletion using Illumina Stranded Total RNA Prep Kit with the Ligation with Ribo-Zero Plus protocol (Illumina). Libraries were sequenced at a depth of 80 million 2 by 100 paired-end reads using an Illumina NovaSeq 6000. Raw sequencing data was preprocessed for adapter removal and quality-based trimming using TrimGalore [10] with removal of autodetected Illumina adapters and trimming of low-quality ends up to a threshold of Q20. Reads that became shorter than 35 basepairs were removed from further analysis. MultiQC was used to assess quality of the reads [11]. Transcript abundances in each sample were quantified using pseudoalignment by Kallisto [12] against the human transcriptome (GRCh38, Ensembl release version 108). *OXTR* expression was measured as transcripts per million.

### DNA Hydroxymethylation Analysis

DNA hydroxymethylation was measured using oxidative bisulfite sequencing with the TrueMethyl oxBS module (Tecan) according to manufacturer instructions. Oxidative bisulfite sequencing which measures true DNA methylation and traditional bisulfite conversion which measures total modified cytosines are completed in parallel [13]. DNA hydroxymethylation is calculated by subtracting true DNA methylation from total modified cytosines. 200 ng genomic DNA was used as input for both the oxidative and traditional bisulfite conversion (with mock oxidation). Following oxidative or traditional bisulfite conversion, PCR was performed in triplicate using 25 ng DNA per reaction. PCR was completed using a Pyromark PCR kit (Qiagen), using the same primers and PCR conditions used in our previous analysis [9]. Pyrosequencing was completed using the Q48 system and reagents (Qiagen). The average of the replicates for both true methylation (average mean deviation of 1.9%) and total modified cytosine (average mean deviation of 1.1%) were used for further analyses. A control sample, which is an in-house generated oligo that is fully hydroxymethylated, was run through all procedures to confirm oxidation efficiency. The oxidized oligo had an average true DNA methylation of 2.26% while the mock-oxidized oligo (which does not differentiate between DNA methylation and hydroxymethylation) has an average DNA methylation of 97.2%, indicating that the oxidative bisulfite procedure effectively differentiates between DNA methylation and hydroxymethylation.

### Statistical Analysis

Analysis of *OXTR* gene expression data was completed using linear models examining an interaction of rs53576 genotype and the epigenetic modification and accounted for RNA quality measured by the RNA Integrity Number (RIN score). Though RIN score was accounted for, *OXTR* expression was not associated with RIN score (Fig. S1a). Analysis of oxytocin administration data was performed by calculating spearman’s rho correlation of *OXTR* DNA methylation and total oxytocin administered. Logistic regression was used to calculate odds for postpartum hemorrhage (estimated blood loss > 1000 mL following birth) with DNA methylation as a predictor, stratified by genotype. We chose to adjust the model by deviation in methylation percentage across pyrosequencing runs as well as parity and total oxytocin exposure (Units) during the labor.

## Results

### DNA Hydroxymethylation and SNP rs53576 Interact to Predict OXTR Expression

**Fig. 1:**
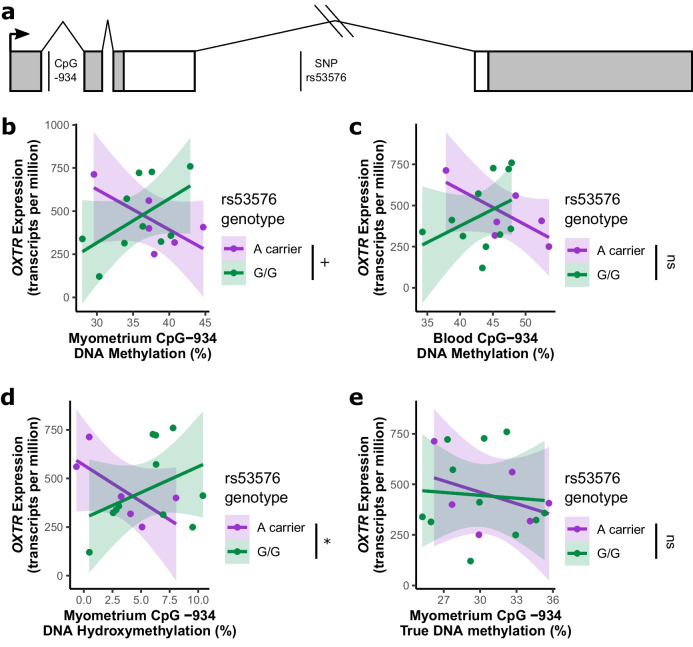
*OXTR* DNA hydroxymethylation and gene expression in pregnant myometrium are positively associated and this relationship is disrupted in rs53576 risk allele carriers. a) Schematic of OXTR indicating where CpG -934 and SNP rs53576 are in the gene. Boxes are exons, lines are introns. Coding regions are in white and untranslated regions are in gray. b) Genotype at rs53576 interacts with CpG -934 methylation to predict *OXTR* expression (*n* = 6 A carriers, 10 G/G genotype, F_(1,11)_ = 4.74, *p* = 0.052). c) Similar genetic by epigenetic interaction based on DNA methylation in blood, though it does not significantly predict gene expression (*n* = 6 A carriers, 11 G/G genotype, F_(1,12)_ = 3.07, *p* = 0.105) d) Genotype at rs53576 interacts with CpG -934 DNA hydroxymethylation to predict *OXTR* expression (*n* = 6 A carriers, 11 G/G genotype, F_(1,10)_ = 5.20, *p* = 0.046). e) True DNA methylation does not interact with rs53576 genotype to predict *OXTR* gene expression (*n* = 6 A carriers, 11 G/G genotype, F_(1,10)_ = 0.69, *p* = 0.504). * *p* < 0.05, + *p* = 0.052, ns *p* > 0.052

In the first cohort, we measured DNA methylation at CpG -934, a well-studied CpG site in the MT2 region of *OXTR*, genotype at SNP rs53576 and *OXTR* expression in pregnant myometrium taken during elective cesarean section (see gene schematic in Fig. 1a). In this cohort, there are eleven individuals with genotype G/G at rs53576, four with genotype A/G, and two with genotype A/A. A/G and A/A individuals are grouped together as “A carriers” for all analyses because of the small sample and number of individuals with these genotypes.

There are no genotype effects on *OXTR* expression in the myometrium (Wilcoxon rank-sum test, W = 32, *p* = 0.961, Fig. S1b), CpG -934 DNA methylation in myometrium (Wilcoxon rank-sum test, W = 39, *p* = 0.368, Fig. S1c), or CpG -934 DNA methylation in blood (Wilcoxon rank-sum test, W = 48, *p* = 0.149, Fig. S1d). Surprisingly, we find that the relationship between CpG -934 methylation and *OXTR* expression depends on rs53576 genotype (ANOVA, rs53576 genotype by CpG -934 DNA methylation interaction F_(1,11)_ = 4.743, *p* = 0.052, Fig. 1b). The typical negative relationship between CpG -934 methylation and *OXTR* expression is present only among A carriers. Conversely, we find a positive correlation among G/G individuals. Similar relationships are present when examining blood-derived DNA methylation, though it is not statistically significant with this small sample, suggesting its utility for making clinical predictions (ANOVA, rs53576 genotype by CpG -934 DNA methylation interaction F_(1,12)_ = 3.07, *p* = 0.105, Fig. 1c), with A carriers being a risk allele.

We reasoned that, given the positive relationship between DNA methylation and *OXTR* expression in G/G women, we are inadvertently measuring a different DNA modification called DNA hydroxymethylation, which cannot be distinguished from DNA methylation using standard assays. DNA hydroxymethylation is an intermediate in the active DNA demethylation pathway which is typically associated with increased gene expression and is known to have important gene regulatory roles in embryonic tissue and neurons [14]. Using a method that measures both DNA hydroxymethylation and true DNA methylation, we find that this genetic by epigenetic interaction occurs specifically for DNA hydroxymethylation (ANOVA, rs53576 genotype by CpG -934 DNA methylation interaction F_(1,12)_ = 5.53 *p* = 0.037; Fig. 1d) and not true DNA methylation (ANOVA, rs53576 genotype by CpG -934 true DNA methylation interaction F_(1,12)_ = 0.161, *p* = 0.694, Fig. 1e). Specifically, in rs53575 G/G individuals, there is the expected, positive relationship between DNA hydroxymethylation and *OXTR* expression. In contrast, A carriers display a negative relationship between DNA hydroxymethylation and *OXTR* expression. Notably, there is no difference in DNA hydroxymethylation or true DNA methylation at CpG -934 by rs53576 genotype (DNA hydroxymethylation: Wilcoxon rank-sum test, W = 21, *p* = 0.256, Fig. S1e; true DNA methylation: Wilcoxon rank-sum test, W = 38, *p* = 0.661, Fig. S1f), indicating that this is a true interaction of genotype and epigenetic state. These results suggest a role for DNA hydroxymethylation in preparing the uterus before birth by upregulating *OXTR* expression. However, this mechanism is altered in rs53576 A carriers, potentially leading to increased risk for birth complications.

### Evidence that a Carriers with High OXTR Methylation Require More Oxytocin During Labor and Have Higher Odds for Postpartum Hemorrhage

**Fig. 2:**
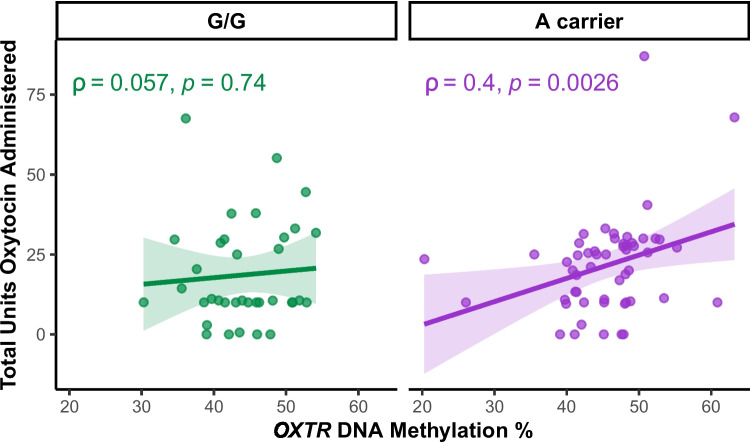
rs53576 A carriers with high *OXTR* methylation in the blood require more oxytocin during labor. In rs53576 A carriers, there is a significant positive relationship between DNA methylation at *OXTR* CpG -934 and total units oxytocin administered during labor (*n* = 57, ρ(55) = 0.4, *p* = 0.0026). In rs53576 G/G individuals, there is no relationship between DNA methylation at *OXTR* CpG -934 and total units oxytocin administered during labor (*n* = 36, ρ(36) = 0.06, *p* = 0.74). Regression lines are shown for visualization purposes

We next re-examined our dataset (cohort 2) indicating that both rs53576 genotype and *OXTR* DNA methylation confer risk for postpartum blood loss [6, 9]. We find that in rs53576 A carriers, there is a significant positive correlation between *OXTR* DNA methylation and amount of oxytocin needed during and after labor, while there is no correlation among G/G individuals (Fig. 2). Since higher *OXTR* methylation is associated with lower *OXTR* expression in rs53576 A carriers only, we suspect that A carriers with high DNA methylation require more units of oxytocin to maintain the labor process because of lower OXTR availability in the myometrium. Notably, many studies report oxytocin administration during labor is a risk factor for postpartum hemorrhage [15]. To this end, the adjusted logistic regression models demonstrated that among A carriers, increasing DNA methylation by 5-percentage points was associated with a doubling of the odds for postpartum hemorrhage (OR 2.07, 95% CI 1.01–4.25, p = 0.048). In contrast, among G/G participants, the odds for postpartum hemorrhage were lower with increasing DNA methylation but the confidence interval was non-significant (OR 0.63, 95% CI 0.06–6.07, p = 0.68). In sum, rs53576 A carriers with high methylation at *OXTR* need administration of more oxytocin and have increased risk of postpartum blood loss, possibly because of reduced *OXTR* expression in myometrium.

## Discussion

Our results provide a mechanism by which a common variant in the oxytocin receptor gene interacts with DNA methylation to confer risk for birth complications resulting from reduced oxytocin signaling. Previously, we have shown that separately the risk allele (A) of rs53576 or higher DNA methylation at *OXTR* increases risk for postpartum hemorrhage [6, 9]. Here, we provide evidence that A carriers of rs53576 with high blood-derived DNA methylation require more oxytocin administration throughout the labor and birth process including administration for labor and/or postpartum uterine atony. A carriers with higher methylation also have higher odds for postpartum hemorrhage. Mechanistically, this may be related to the relationship between DNA hydroxymethylation and *OXTR* gene expression in the myometrium. Prior to birth, we propose that DNA methylation of *OXTR* is converted to DNA hydroxymethylation to increase *OXTR* expression. In rs53576 G/G individuals, this DNA hydroxymethylation does lead to increased *OXTR* expression, but in A carriers, it does not. Thus, risk conferred by the A allele of rs53576 might be due to the inability of this allele to respond to DNA hydroxymethylation and modulate gene expression.

We do not yet know why A carriers are unable to respond to DNA hydroxymethylation, but it could be because of differences in local chromatin state or transcription factor binding. Further work examining how these factors are related to SNP rs53576 would clarify these results. It remains possible that rs53576 is not the “causative” SNP but is actually in linkage disequilibrium with the SNP that is functionally responsible for mediating this described interaction; furthermore, the functional SNP might be closer to CpG -934, making more mechanisms for the interaction plausible.

Though our sample is small, our preliminary evidence indicates that the genetic by epigenetic interaction predicting *OXTR* gene expression in myometrium may be present when measuring DNA methylation from blood. This may allow for use of blood-based measurements of rs53576 genotype and DNA methylation to identify women at high risk of oxytocin-related birth complications. Indeed, we provide evidence that in A carriers, higher DNA methylation in blood (which my correlate with lower *OXTR* expression in myometrium) is associated with higher clinical oxytocin needs and hemorrhage risk. We note that while we describe a similar genetic by epigenetic interaction predicting *OXTR* expression in myometrium using blood-derived DNA methylation, we did not measure DNA hydroxymethylation in blood because of its extremely low abundance in blood tissue [16]. Nonetheless, we believe our findings warrant following up in a larger sample to determine if blood-derived DNA methylation combined with rs53576 genotype is clinically informative of risk of birth complications, possibly because it reflects *OXTR gene expression in myometrium.*

This study is limited in a few ways. First, the gene expression study (Fig. 1) is completed in a small sample which was originally not collected for gene expression studies. RNA quality of these samples was not ideal, though RNA quality did not correlate with *OXTR* expression (Fig. S1a) and was accounted for in statistical models. A replication study in a larger sample with higher RNA quality should be performed to replicate these results. Though we suspect that DNA hydroxymethylation accumulates at *OXTR* in myometrium over the course of pregnancy to increase *OXTR* expression, our study is limited by only collecting term pregnant myometrium. Testing this hypothesis would require acquiring myometrium tissue at several timepoints throughout gestation, possibly necessitating an animal model. Finally, we did not measure OXTR protein abundance in these samples, which is necessary to establish that functional regulation of *OXTR* gene expression has consequences for OXTR protein levels in pregnant myometrium, ultimately allowing for a mechanistic understanding of how *OXTR* epigenetic regulation relates to myometrial contraction and responsiveness to oxytocin. Nonetheless, these results represent a significant advance in our understanding of how genetic variation in *OXTR* might lead to oxytocin sensitivity during pregnancy and postpartum hemorrhage. Further, we demonstrate how the epigenetic modification DNA hydroxymethylation regulates uterine physiology. Future studies that replicate these results would allow for identification of women at risk for postpartum hemorrhage and other birth complications using blood-derived DNA methylation measurements and genotyping.

## Supplementary Information

Below is the link to the electronic supplementary material.

Supplementary file1 (DOCX 123 KB)

## Data Availability

Data is available upon reasonable request to the corresponding author. For data requests as part of extension to this study, data may be limited to participants who consented to data use in future research during the informed consent process.
