# Supplementary material for: A Common OXTR Risk Variant Alters Regulation of Gene Expression by DNA Hydroxymethylation in Pregnant Human Myometrium
Source: Reprod Sci. 2024 Jun 11;31(10):3132–8. doi: 10.1007/s43032-024-01621-9 (PMC11438727; doi:10.1007/s43032-024-01621-9)
Supplement: Supplementary file 1 — Supplementary file1 (DOCX 123 KB) [file 43032_2024_1621_MOESM1_ESM.docx]

**
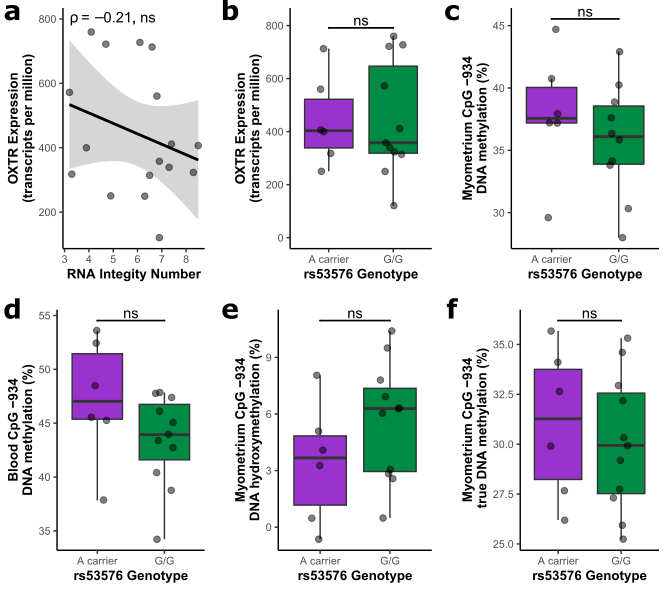
**

**Fig. S1. *OXTR* expression and CpG -934 epigenetic modifications are not associated with rs53576 genotype.** a) *OXTR* expression and sample quality (RNA integrity number) are not correlated (Spearman’s ρ=-0.212, *p*=0.403). A regression line is shown for visualization purposes. b) rs53576 genotype does is not associated with *OXTR* gene expression in myometrium (Wilcoxon rank-sum test, W=32, *p*=0.961). c) rs53576 genotype is not associated with CpG -934 DNA methylation in myometrium (Wilcoxon rank-sum test, W=39, *p*=0.368). d) rs53576 genotype is not associated with CpG -934 DNA methylation in blood (Wilcoxon rank-sum test, W=48, *p*=0.149). e) rs53576 genotype is not associated with DNA hydroxymethylation at CpG -934 in myometrium (Wilcoxon rank-sum test, W=21, *p*=0.256). f) rs53576 genotype is not associated with true DNA methylation at CpG -934 in myometrium (Wilcoxon rank-sum test, W=38, *p*=0.661).
